# Characteristics of Suicide Attempts in Anorexia and Bulimia Nervosa: A Case–Control Study

**DOI:** 10.1371/journal.pone.0023578

**Published:** 2011-08-12

**Authors:** Sébastien Guillaume, Isabelle Jaussent, Emilie Olié, Catherine Genty, Jacques Bringer, Philippe Courtet, Ulrike Schmidt

**Affiliations:** 1 Inserm, U1061, Montpellier, France; 2 Université Montpellier I, Montpellier, France; 3 CHU Montpellier, Hôpital Lapeyronie, Psychiatric Emergency and Post Emergency Department, Pole Urgence, Montpellier, France; 4 King's College London, Institute of Psychiatry, London, United Kingdom; 5 Endocrinology Department, CHU Montpellier, Montpellier, France; The University of Queensland, Australia

## Abstract

**Objective:**

Compared to other eating disorders, anorexia nervosa (AN) has the highest rates of completed suicide whereas suicide attempt rates are similar or lower than in bulimia nervosa (BN). Attempted suicide is a key predictor of suicide, thus this mismatch is intriguing. We sought to explore whether the clinical characteristics of suicidal acts differ between suicide attempters with AN, BN or without an eating disorders (ED).

**Method:**

Case-control study in a cohort of suicide attempters (n = 1563). Forty-four patients with AN and 71 with BN were compared with 235 non-ED attempters matched for sex, age and education, using interview measures of suicidal intent and severity.

**Results:**

AN patients were more likely to have made a serious attempt (OR = 3.4, 95% CI 1.4–7.9), with a higher expectation of dying (OR = 3.7,95% CI 1.1–13.5), and an increased risk of severity (OR = 3.4,95% CI 1.2–9.6). BN patients did not differ from the control group. Clinical markers of the severity of ED were associated with the seriousness of the attempt.

**Conclusion:**

There are distinct features of suicide attempts in AN. This may explain the higher suicide rates in AN. Higher completed suicide rates in AN may be partially explained by AN patients' higher desire to die and their more severe and lethal attempts.

## Introduction

Eating disorders (ED) are biologically based serious mental disorders with high levels of mortality and disability, physical and psychological morbidity and impaired quality of life [1]. In anorexia nervosa (AN), this excess mortality is explained in part by the physical complications and in part by an increased rate of suicide. Across studies, approximately 20 to 40% of deaths in AN are thought to result from suicide [2], [3] with SMRs for suicide of 31 in a recent meta analysis [4] and ranging from 13.6 to 56.9 [2], [3], [5]. In bulimia nervosa (BN), the same meta-analysis found a lower SMR of 7.5 [4] with some studies showing no excess mortality [6] and more recent data suggesting that suicide rates may be increased in this group [7].

Rates of suicidal and self-harming behaviors in ED populations are also raised compared to healthy controls [8], [9] and comparable or higher than in other psychiatric populations [9], [10]. Studies comparing rates of suicidal behavior in AN and BN have had mixed results with some finding no difference between AN and BN [10], [11] and others finding lower rates of suicide attempts (SA) in AN than BN [12] [for review see [13]].

In general, attempted suicide is one of the most potent and reliable predictors of completed suicide [14], thus the mismatch between attempted and completed suicide in different ED diagnoses and subtypes is intriguing. What explains this discrepancy? One possibility is that as people with AN are more physically compromised than those with BN, this may make them more likely to die from a suicide attempt. Another possibility is that people with AN may make more severe and lethal attempts than those with BN, perhaps as a result of differences in the motives for suicidal behavior due to underlying personality traits or axis I or II comorbidity. In line with this hypothesis, a case series of nine completed suicides in AN patients found that the majority of these deaths were caused by use of methods with low rescue potential and high likelihood of death (e.g. jumping in front of a train or hanging). Nevertheless, to date no study has compared eating-disordered suicide attempters with a comparison group of suicide attempters who do not have an ED with respect to the characteristics of their suicide attempt. This comparison potentially has important clinical implications.

### Aims of the study

The aim was to determine whether in a prospectively gathered and well-characterized cohort of current suicide attempters the clinical characteristics of suicidal acts differ between suicide attempters with life-time or current AN or BN and suicide attempters without ED. We hypothesized that AN patients would show SA that are more severe than those in BN and other non-eating disordered attempters. We also hypothesized that suicide attempts by people with BN would be broadly similar to attempts in a general population of suicide attempters.

## Methods

### Participants and clinical assessment

Study participants were identified from a large cohort of suicide attempters (n = 1563), consecutively hospitalized and survivors of a current suicide attempt (SA) in a specialized unit of the Montpellier University Hospital. Patients included in the cohort had to be between 18 and 75 years old, French-speaking, and with all four biological grandparents originating from Western European countries (for genetic purposes). This cohort is part of a large ongoing multi-centre study of suicidal behavior, which has been approved by the local research ethics committee (Comité de protection des personnes “Sud-Méditerranée IV” Lapeyronie hospital, Montpellier France). After having received information on the study, potential participants completed and returned a consent form.

**Figure 1:**
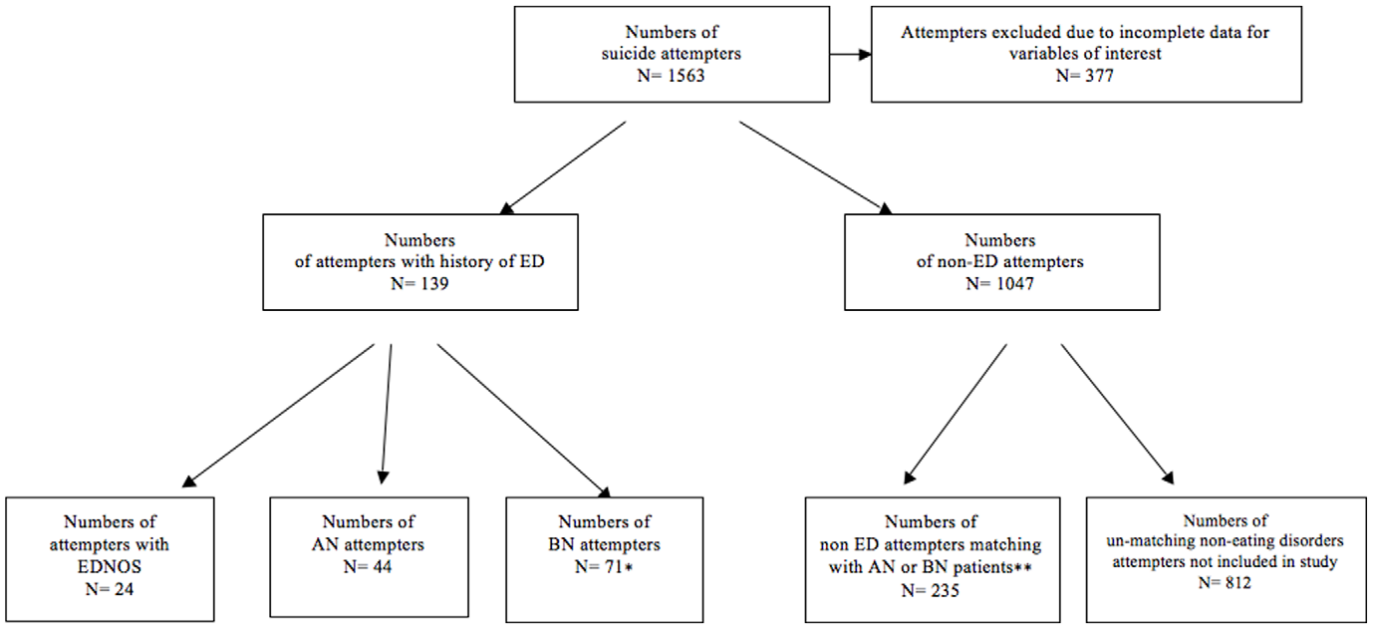
Participant selection procedure.

In total, 139 patients with a lifetime history of DSM-IV eating disorders were identified, constituting 11.2% of the total cohort. Of these, 44 patients satisfied the lifetime criteria for anorexia nervosa (of whom twenty-eight (63.6%) had a current ED at the time of their attempt), 64 fulfilled the lifetime criteria for bulimia nervosa (of whom fifty-two (73.2%) BN patients had a current ED at the time of their attempt), 7 met both the lifetime criteria for AN and BN and 24 for Eating Disorders Not Otherwise Specified (EDNOS). Patients were split into two groups, a lifetime AN (n = 44) and a BN group which included those with lifetime BN and both lifetime BN and AN (n = 71), as in these cases BN was the most recent diagnosis before the SA. Patients with EDNOS were excluded due to their low number and the heterogeneity of this group. A control group of 235 SA patients without an ED and matched for age (± one year), sex and education level was also selected from the larger cohort of suicide attempters. The selection procedure of participants for the present study is described in figure 1.

Patients were evaluated after remission of a potential mood episode (i.e. a current Hamilton Depression Rating Scale score below 15). They were interviewed by trained psychiatrists or psychologists, using the Mini International Neuropsychiatric Interview (MINI) [15]. Current and lifetime DSM-IV diagnoses (including ED diagnoses) were assessed by the interviewer and then blindly rated by an independent psychiatrist according to medical case notes and MINI. Further information about ED history was obtained for each patient using their medical records and when available, information from relatives. The Diagnostic Interview for Genetic Studies and dimensional scales (see below) were used to obtain information regarding suicidal behavior.

Suicide attempts were defined as the occurrence of self-directed injurious acts with intent to end one's own life [16]. As previously described [17], a serious attempt was defined as an attempt which was deemed to be violent (i.e. using method other than poisoning or wrist cutting such as hanging, drowning, using a firearm…) [18] or one that justified admission to an medical intensive care unit.

For participant with a history of multiple suicide attempts, only the most severe suicide attempt was considered for analysis (which was also the current SA for 34.2% of the sample). The most severe suicide attempt was defined as the attempt with the most severe medical consequences and thus the highest score on the risk section of the Risk Rescue Rating Scale (see below).

We characterized this most severe SA using the French versions of three observer-rated scales: (1) The Risk Rescue Rating Scale (RRRS) [19]: this is a 10-item scale, with 5 items evaluating the risk of the suicidal act (primarily the medical consequences) (RRRS risk scores range from 5 to 15; a high score denotes high risk) and 5 items evaluating the likelihood of rescue following an attempt (RRRS rescue scores range from 5 to 15; a high score denotes high rescue potential). (2) The Suicidal Intent Scale [20]: this is a 15-item scale designed to assess the severity of suicidal intention associated with a SA. This scale provides a total score consisting of two sub-scores, one assessing the objective circumstances of the attempt (SIS planning: scores range from 0 to 16; a high score denotes high planning), the other assessing the belief the person had about the severity of the attempt (SIS conception: score from 0 to 14; a high score denotes a higher expectation of dying). (3) The scale for suicide ideation (SSI) [21]: this is a 21-item rating scale that measures the intensity of person's suicidal ideation (scores range from 0 to 38; a high score denotes a high level of suicide ideation). This scale was retrospectively rated to assess the period just before the attempt.

### Statistical analysis

The sample is described using percentages for categorical variables and medians and ranges for continuous variables (age at diagnosis, age at onset of first SA) as their distributions were tested with the Shapiro-Wilk statistic and were skewed. Clinical and social characteristics between cases and controls were compared using Chi-square tests (for categorical variables) or Mann-Whitney tests (for continuous variables).

Univariate comparisons between the cases and controls were performed using unconditional logistic regression models including stratification variables, i.e. gender, age and educational level. Finally, Spearman's rank-order correlations were used to measure the associations between two continues variables. Significance was set at p<0.05. Statistical analyses were carried out using SAS version 9.1 (SAS Institute, Inc. Cary, North Carolina).

## Results

### Demographics and clinical characteristics

Table 1 shows the sociodemographic characteristics and comorbid DSM-IV lifetime axis I diagnoses of the whole ED sample and the control sample. No significant difference was found between the groups regarding lifetime comorbid axis-I disorders. Interestingly, among ED patients only one (an AN patient) was free of any comorbid diagnosis. Finally, there were no significant differences between the group in proportion of patient with a chronic medical comorbidity (i.e: diabetes mellitus, thyroid disorders or chronic neurologic disorders such as epilepsy).

**Table 1:** Sample description for the eating disorder and control participants.

	*Controls* *N = 235*		*EDs* *N = 139*		
*Variable*	*n*	*%*	*n*	*%*	*P-value*
Gender^a^					
Male	11	4.7	6	4.3	0.87
Female	224	95.3	133	95.7	
Median age at the inclusion (in years)[Range]^a^	33.8 [18.1–60.7]		31.0 [18.0–60.1]		0.46
Education Level^a^					
<9years	48	20.4	30	22.1	0.76
10–12 years	99	42.1	52	38.2	
>12 years	88	37.5	54	39.7	
Living with a partner					
No	168	71.5	96	69.6	0.69
Yes	67	28.5	42	30.4	
Bipolar disorder^b^^,^ c					
No	180	76.6	101	73.2	0.46
Yes	55	23.4	37	26.8	
Major depressive disorder^b^					
No	64	27.2	40	28.8	0.75
Yes	171	72.8	99	71.2	
Anxiety disorder^b^					
No	67	29.1	37	26.8	0.63
Yes	163	70.9	101	73.2	
Substance use disorder^b^					
No	196	83.4	112	80.6	0.49
Yes	39	16.6	27	19.4	
Alcohol use disorder^b^					
No	175	74.5	105	76.1	0.73
Yes	60	25.5	33	23.9	
Smoking					
No	59	25.1	27	19.6	0.44
Currently	146	62.1	94	68.1	
In the past	30	12.8	17	12.3	
Chronic diseases					
No	195	84.42	107	79.85	0.27
Yes	36	15.58	27	20.15	

a Matching variables,

b Life time diagnoses,

c non significant difference between bipolar disorders type 1 and type 2.

ED = eating disorders.

Table 2 shows clinical characteristics and lifetime comorbid DSM-IV diagnoses for the whole ED sample and ED subgroups. The age of onset of ED in AN and BN patients did not differ. As expected in the ED sample, the lowest body mass index (BMI) since onset was lower in the AN sample than in the BN sample (p<0.0001). The proportion of patients with a previous hospitalization was higher in the AN sample than in BN sample (p<0.0001). No significant differences were found between the AN and BN groups in terms of comorbid axis-I disorders.

**Table 2:** Description of the eating disorder sample.

	*EDs* *N = 139*	*AN* *N = 44*	*BN* *N = 71*	p-value
Age at ED onset, Median [range]	(n = 122) 17 [10–55]	(n = 42) 17 [10–45]	(n = 59) 17 [10–45]	0.62
Lowest BMI since onset of ED, Median [range]	(n = 126)17.6 [9.0–33.7]	14.85 [9.0–17.3]	19.8 [10.3–33.7]	<0.0001
History of previous hospitalisation for ED, n (%)	(n = 122) 25 (19)	20 (45.0)	5 (8.2)	<0.0001
Time between onset of ED and first SA (years), Median [range]	(n = 122) 1 [−24–32]	(n = 42) 3 [−15–25]	(n = 59) 0 [−20–32]	0.10
Time between onset of ED and current SA (years), Median [range]	(n = 122) 8.7 [0.4–37.5]	(n = 42) 10.10 [0.9–35.6]	(n = 59) 8.0 [0.4–37.5]	0.48
Chronic diseases	27 (20.2)	10 (24.4)	12 (17.1)	0.36
Bipolar disorder*, n (%)	37 (26.8)	14 (31.8)	16 (22.9)	0.29
Major depressive disorder*, n (%)	99 (71.2)	29 (65.9)	53 (74.6)	0.31
Anxiety disorder, * n (%)	101 (73.2)	30 (68.2)	50 (71.4)	0.71
Substance use disorder*, n (%)	27 (19.4)	9 (20.5)	15 (21.1)	0.93
Alcohol use disorder*, n (%)	33 (23.9)	11 (25.6)	18 (25.4)	0.98
Tobacco use*, n (%)	111 (80.4)	34 (77.4)	60 (84.5)	0.33

*Life time diagnose.

ED = eating disorders, AN = Anorexia nervosa, BN = Bulimia nervosa, SA = Suicide attempt.

### Characteristics of the Suicidal Behavior

Table 3 shows characteristics of suicidal behavior in non-ED controls, ED patients and sub-groups (AN, BN). Comparing controls and the whole ED sample, a higher proportion of ED patients had a lifetime history of a serious SA (OR 1.8; CI 1.1 to 3.0) and a higher proportion of ED patients had a history of recurrent SA (i.e. at least two SA) (OR = 1.9; CI 1 to 2.9).

**Table 3:** Characteristics of suicidal behavior in ED patients and controls.

	*Controls* *N = 235*	*ED Cases* *N = 139*			*AN* *N = 44*			*BN* *N = 71*		
*Variable*	*%*	*%*	*OR [95% CI]* *	*P-value*	*%*	*OR [95% CI]* *	*P-value*	*%*	*OR [95% CI]* *	*P-value*
History of familial suicide behavior										
No	54.4	56.4	1	0.76	59.5	1	0.28	55.7	1	0.87
Yes	45.7	43.6	0.9 [0.6–1.5]		40.5	0.6 [0.3–1.4]		44.3	0.9 [0.5–1.8]	
Type of SA lifetime										
Not serious	73.0	63.1	1	0.0153	52.3	1	0.0054	67.6	1	0.34
Serious	27.0	36.9	1.8 [1.1–3.0]		47.7	3.4 [1.4–7.9]		32.4	1.4 [0.7–2.9]	
Age at first SA	24 [7–56]	21 [10–57]	0.8 [0.6–1.1]	0.19	21.5 [12–56]	0.8 [0.5–1.1]	0.14	21 [10–54]	1.0 [0.6–1.6]	0.93
Number of SA										
1	34.1	24.3	1	0.0393	27.9	1	0.60	25.7	1	0.17
2 or more	65.9	75.7	1.7 [1.0;2.9]		72.1	1.3 [0.5;3.1]		74.3	1.7 [0.8; 3.4]	
RRRS risk										
<7	32.0	25.2	1	0.22	18.6	1.0 [0.3;3.3]	0.0451	28.1	1	0.54
7–9	34.2	32.0	1.2 [0.6;2.1]		30.2	1		31.3	1.5 [0.6;3.3]	
≥9	33.8	42.8	1.7 [0.9;2.9]		51.2	3.4 [1.2;9.6]		40.6	1.5 [0.7;3.4]	
RRRS rescue										
<12	27.3	24.4	1	0.42	15.8	1	0.52	31.2	1	0.24
12–14	40.7	34.4	1.0 [0.5;1.7]		47.4	1.8 [0.6;5.0]		26.2	0.5 [0.2;1.2]	
> = 14	32.0	41.2	1.4 [0.8;2.5]		36.8	1.8 [0.6;5.4]		42.6	1.0 [0.4;2.2]	
SIS planning										
≤4	33.6	38.0	1	0.45	35.71	1	0.90	39.1	1	0.71
4–7	33.2	31.0	0.9 [0.5;1.5]		28.57	0.9 [0.4;2.3]		31.2	0.7 [0.3;1.6]	
>7	33.2	31.0	0.7 [0.4;1.2]		35.71	0.8 [0.3;1.9]		29.7	0.9 [0.4;2.0]	
SIS conception										
<8	35.5	25.8	1	0.33	17.1	1	0.0998	32.8	1	0.28
8–12	32.0	38.3	1.6 [0.9;2.8]		34.2	1.8 [0.5;6.4]		35.9	1.6 [0.7;3.5]	
≥12	32.5	35.9	1.3 [0.8;2.4]		48.8	3.7 [1.1;13.5]		31.3	0.8 [0.4;1.8]	
SIS total										
≤12	35.5	29.8	1	0.87	21.95	1	0.51	37.70	1	0.87
12–18	34.7	36.3	1.2 [0.7;2.0]		36.59	1.9 [0.6;5.8]		32.79	0.9 [0.4;1.9]	
>18	29.8	33.9	1.1 [0.6;2.0]		41.46	1.3 [0.4;4.1]		29.51	1.1 [0.5;2.6]	
SSI total										
<20	34.9	27.3	1	0.12	26.3	1	0.43	28.6	1	0.26
20–26	31.1	37.2	2.0 [1.0;3.7]		28.9	1.9 [0.6;6.4]		38.1	2.0 [0.8;5.0]	
≥26	33.9	35.5	1.4 [0.8;2.6]		44.8	1.0 [0.3;3.0]		33.3	1.8 [0.7;4.1]	

*Odds ratio (OR) were derived from an unconditional logistic model, adjusted for age, gender and educational level.

ED = eating disorders, AN = Anorexia nervosa, BN = Bulimia nervosa, SA = Suicide attempt, RRRS = Risk Rescue Rating Scale, SIS = Suicide Intent Scale, SSI = Scale for Suicide Ideation.

Compared to controls, AN patients were significantly more likely to have made a serious SA (OR = 3.4; 95% CI 1.4 to 7.9). They also had a higher risk of having made a highly severe SA as assessed by the RRRS-risk scale (OR = 3.4; 95% CI 1.2 to 9.6) and their most severe SA was characterized by a higher expectation of dying on the SIS (OR = 3.7; 95% CI 1.1 to 13.5).

The BN group was not significantly different to the control group of non-ED suicide attempters for any of the above characteristics of their SA.

We also compared the characteristics of suicidal behavior between the BN and AN group. Serious suicide attempts tended to be occur more commonly in AN (OR = 2.2 95% CI = [1.0 to 5.0], p = 0.0585) and expectation of dying (SIS conception score) was associated with AN (OR = 3.2 95%CI = 1.1 to 9.5 for the last tercile, p = 0.0354).

Twenty-eight (63.6%) AN and fifty-two (73.2%) BN patients had a current eating disorder at the time of their attempt. We performed a sensitivity analysis in people with a current eating disorder only (i.e. excluding people with a remitted eating disorder) and the results remain the same. The only exception was the “expectation to die” (SIS conception score) which was not significantly different between current AN patients and controls (OR = 5.0; CI 0.9 to 27.7).

A history of hospitalization for an ED was associated with a higher lifetime history of a serious SA (OR = 4.0; CI 1.5 to 10.5) and during their most severe attempt a higher RRRS rescue score (OR = 6.4; CI 1.2 to 33.4) suggesting a higher likelihood of rescue during their most severe attempt. None of the other SA features were different. (Data not shown).

Finally, we compared patients with a lifetime binge-purging subtype (irrespective of the diagnosis of AN or BN) versus patients with a lifetime restrictive subtype only. 74% (n = 85) of the ED patients had a purging subtype and 26% (N = 30) a restrictive subtype. Patients with a purging subtype were younger when they made their first SA (median age of 21 vs 24.5 years; p = 0.03). There were no differences between purging and restricting sub-type patients for the other features of the SA.

### Correlations between clinical variables

We performed correlations in the ED group only. The lowest BMI since ED onset was negatively correlated with the “expectation to die” (r = −0.29, p = 0.004) and with the intensity of suicidal ideation before the attempt (SSI total score) (r = −0.26, p = 0.013). There were no other significant correlations between clinical characteristics (lowest BMI, age of ED onset, time between onset of ED and first SA, time between onset of ED and most lethal SA and time between onset of ED and current SA) and suicidal scales.

## Discussion

The main aim of our study was to assess the clinical features of suicidal acts in people with AN or BN compared to suicide attempters without an ED, and compared to each other. Our study is the first to show conclusively that AN patients make more serious and severe SA and have a higher expectation that they will die from their attempt than other suicide attempters. In contrast, the features of SA in BN patients seem very similar to those of suicide attempters without ED.

Previous studies have suggested that AN patients who make a suicide attempt have high suicidal intent [10], [22], however, our study is the first to note the greater severity of SA in this group compared to non-ED groups. Earlier studies have suggested that comparative suicide rates in AN may be inflated due to reliance on AN in-patient samples [23]. In the present study, only a sub-group of AN had a history of hospitalization, thus countering this idea.

Although our study concerned suicide attempters and not patients with completed suicide, our findings could also help to explain the intriguing discrepancy between rates of SA and completed suicides in AN and BN. Higher completed suicide rates in AN in spite of equivalent or lower rates of SA compared to BN may at least partially be explained by AN patients' higher desire to die and their more severe attempts. Our findings are congruent with those from a case series of nine completed suicides in AN patients which found that the majority of these deaths were caused by use of methods with low rescue potential and high likelihood of death (e.g. jumping in front of a train or hanging) [24]. This suggests that deaths from suicide in AN are not usually the result simply of their greater physical frailty compared to other suicide attempters.

We can only speculate on the mechanisms underlying the greater seriousness of AN patients' suicide attempts. Recent theories of suicidal behavior such as the Interpersonal-Psychological theory of suicidal behavior [25] or the Stress-Diathesis model of suicidal behavior [16] may help to understand the higher lethality of SA in AN compared to other suicide attempters. Joiner's theory posits that in general suicide results when three factors combine: (a) a feeling of being alone and not belonging; (b) a sense of being a burden to others, and (c) an acquired ability to endure pain. AN is a chronic and often severe disorder with poor quality of life, social isolation, loneliness and burdensomeness to self and others [26], [27]. Increased ability to endure pain arises through either extreme food restriction or regular exposure to behaviors such as vomiting, laxative abuse and self-injury [23]. The unique interplay between these predisposing social evaluative and physical factors in people with AN may explain the greater seriousness of SA in this group compared to other suicide attempters. In terms of Mann's stress diathesis model, previous studies in ED have shown that attempts are linked with factors known to increase diathesis to suicidal behavior in general [16], such as impulsive [11] and anxious personality traits [10], [28], [29] or childhood trauma, such as sexual abuse [13]. Unique stressors triggering SA in AN may be starvation related increases in depressive symptomatology and associated cognitive impairments. Unfortunately, we did not assess patients' BMI patients at the time of their SA, but this latter suggestion is supported by the fact that in our sample lowest BMI since ED onset correlated with the belief that the SA would cause death and the intensity of suicidal ideation before the attempt. Moreover, we found that previous hospitalization, i.e. a marker of clinical severity of the ED was associated with severity of SA.

In this context, a particular finding was that whilst patients with a history of hospitalization for an ED had a higher lifetime history of a serious SA than those without hospitalization, they also had a higher likelihood of rescue during their most severe attempt. One potential explanation is that this reflects greater willingness to seek help amongst this subgroup.

Another important factor that might be at play is psychiatric comorbidity. Until now, most of the studies focusing on SA in ED have studied characteristics of ED with or without history of SA. One of the stronger correlates of SA usually found is psychiatric comorbidity (particularly mood disorders). The literature suggests that bipolar depressive patients have a much higher risk of completed suicide [30] and a higher rate of suicide attempts [31] than unipolar ones. Although the difference between major depressive disorder and bipolar disorder among our AN and BN patients is not significant, AN patients had higher rate of bipolar disorder and BN patients had higher rate of major depressive disorder. These differences in term of mood disorders comorbidity rates may also play a role in the greater seriousness of AN patients' suicide attempts.

Our study has considerable methodological strengths. The sample is reasonably large given the low prevalence of disorders like AN. The patients were carefully assessed using structured and well-validated instruments. The control group was matched in term of sex, age and education. Furthermore, to the best of our knowledge, this study is the first to assess SA characteristics in different ED among a sample of suicide attempters.

### Limitations

Limitations include firstly, that we did not assess patients' BMI patients at the time of their SA. This prevents us from determining how BMI relates to risk of SA. We also cannot exclude the possibility that the higher lethality of the SA in the anorexic sample was a consequence of a poorer physical state. Against this is the fact that AN patient reported having a high expectation of dying before their attempt. The expectation that one will die as a result of the SA, might be a better index of the seriousness of a SA than medical severity [32] because attempters are often unaware of the potential lethality of a drugs or a method. Secondly, SA was assessed retrospectively. However, it is unlikely that ED and controls patients would differ in their degree of recall bias for the variables examined. Thirdly, we did not assess comorbid personality disorders. Lastly, we studied only those patients who were admitted to our unit (the only one dealing with suicide attempters in the area) after an initial assessment in one of several emergency rooms of the city. Thus, we did not include (and have no information) on patients who were discharged after initial assessment and treatment in the emergency room. Hence, our results are generalisable to patients admitted to a specialized unit following a suicide attempt.

Higher completed suicide rates in AN in spite of equivalent or lower rates of SA compared to BN may at least partially be explained by AN patients' higher desire to die and their more severe and lethal attempts. Clinicians need to be alert to the high risk of lethal SA among patients with current or past AN. A careful and repeated evaluation of suicidal risk in these patients could improve the early detection and treatment of suicidal behavior. Recent data suggest an elevated suicide rate in EDNOS patients [7]. Our study did not include people with EDNOS, but future studies should focus on this patient group. Future studies should also focus on developing a better understanding of the mechanisms which specifically increase the suicide risk in AN.
